# Prevalence of stunting and its correlates among children under 5 in Afghanistan: the potential impact of basic and full vaccination

**DOI:** 10.1186/s12887-024-04913-w

**Published:** 2024-07-06

**Authors:** Omid Dadras, Charuai Suwanbamrung, Massoma Jafari, Muhammad Haroon Stanikzai

**Affiliations:** 1 Department of Global Public Health and Primary Care, University of Bergen, Bergen, Norway; 2 Public Health Research Program, School of Public Health, Walailak University, Thai Buri, Thailand; 3 Excellent Center for Dengue and Community Public Health (EC for DACH), Walailak University, Thai Buri, Thailand; 4 McMaster University, Hamilton, ON Canada; 5 Department of Public Health, Faculty of Medicine, Kandahar University, District # 10, Kandahar, 3801 Afghanistan

**Keywords:** Children under 5; stunting, Afghanistan, Prevalence, Correlates

## Abstract

**Background:**

Child stunting is prevalent in low and middle-income countries (LMICs), but an information gap remains regarding its current prevalence, correlates, and the impact of vaccination against this condition in Afghanistan. This study aimed to determine the prevalence and correlates of moderate and severe stunting and the potential impact of basic and full vaccination among children under five in Afghanistan.

**Methods:**

This is a secondary analysis of the 2022-23 Afghanistan Multiple Indicators Cluster Survey (MICS) including 32,989 children under 5. Descriptive statistics were employed to describe the distribution of independent variables and the prevalence of stunting across them. Chi-square analysis was used to examine the association between each independent variable with stunting. Multinomial logistic regression was used to examine the risk of stunting across different independent variables.

**Results:**

A total of 32,989 children under 5 years old were included in this study. Of those 44.7% were stunted with 21.74% being severely stunted. Children aged 24–35 and 36–47 months faced the highest risk as compared to those aged 1–5 months. The prevalence was lower in female children and they were less likely to experience severe stunting. Stunting was more prevalent in rural areas, with children there 1.16 to 1.23 times more likely to be affected than urban counterparts. Lower wealth correlated with higher stunting. Younger maternal age at birth (≤ 18) correlated with increased stunting risks, particularly in severe cases. Parental education was inversely related to stunting; higher education levels in parents, especially fathers, were associated with lower stunting rates. Households with more than seven children showed a 25% and 44% higher risk of moderate and severe stunting, respectively, compared to families with 1–4 children. Improved sanitation, but not drinking water sources, was linked to reduced stunting in the adjusted model. Vaccination had a protective effect; in the adjusted analysis, basic and full vaccinations significantly lowered the risk of severe stunting by 46% and 41%, respectively.

**Conclusion:**

In this nationally representative study, the prevalence of stunting was substantial (44.7%) in Afghan children. Additionally, the findings emphasize the critical factors associated with child stunting and underscore the protective role of vaccination against this condition, which provides policymakers with directions for policy efforts and intervention strategies to reduce child stunting in Afghanistan.

## Introduction

Stunting, characterized by impaired growth and development in children due to poor nutrition and recurrent infections, remains a significant public health challenge worldwide. In 2022, 148.1 million children under 5 years of age were affected by stunting globally [1]. It is particularly more prevalent in low-income countries with an estimated mean prevalence of 34.6% [2]. The consequences of child stunting are both immediate and long-term, including increased morbidity, poor child development and learning capacity, increased risk of infections and non-communicable diseases, and lower economic potential [3]. Despite concerted global efforts, progress in reducing child stunting has been uneven, with some regions lagging notably behind others [4]. In 2022, nearly two out of five children with stunting lived in South Asia, while another two out of five lived in sub-Saharan Africa [4].

In Afghanistan, the situation is particularly dire. Four decades of political and economic instability, compounded by natural disasters, poverty, and inadequate healthcare access, have entrenched child health (including stunting) in this country [5]. The 2013 Afghanistan National Nutrition Survey revealed significant nutritional challenges among children under five: 46.2% were stunted, 11.6% wasted, 24.4% underweight, and 19.9% overweight or obese [6]. Although there has been some improvement since 2013, the high prevalence of undernutrition, stunting, and wasting calls for immediate intervention to address these persistent issues [7]. Health service coverage has seen a 30% improvement since 2010, achieving a 37% coverage rate, yet a substantial proportion of children are not fully vaccinated, with 60% and 33% lacking complete measles and diphtheria-tetanus-pertussis immunizations respectively [8] This shortfall in immunization leaves them vulnerable to infections, which can precipitate or exacerbate conditions like stunting [9].

The maternal and child health crisis in Afghanistan is significantly worsened by high maternal, neonatal, and under-five child mortality rates, with 638 per 100,000 live births and 36 and 60 per 1,000 live births, respectively [10, 11]. A substantial portion of these child deaths are attributable to preterm births and infectious diseases [12]. Contributing factors include increased urbanization, dietary shifts, and over one-third of Afghan women lacking access to skilled birth attendants during childbirth [12]. UNICEF’s 2021 report indicates that approximately 41% of children under 5 in Afghanistan are stunted, indicating a sign of chronic undernutrition [13]. The situation appears increasingly dire as UNICEF’s 2023 report anticipates a critical need for intervention, projecting that 875,000 children under five will require treatment for severe acute malnutrition, as detailed in the Humanitarian Needs Overview for 2023 [14]. Furthermore, the health system faces mounting pressures from the recent economic decline, attributed to the COVID-19 pandemic and exacerbated by sanctions and funding restrictions on the political transition since 2021 [15]. These factors have disrupted essential healthcare services (including vaccination campaigns), aggravated malnutrition, increased food scarcity, and healthcare challenges for children.

The need for this research arises from the critical gap in understanding the current prevalence and correlates of child stunting within the Afghan context. It is more critical for the potential impact of child vaccination given the lag that is caused by the current sociopolitical situation. While global studies provide a broad picture, detailed, potential context-specific interactions are scarce for Afghanistan in recent years. This research aims to fill this gap by examining the prevalence and correlates of moderate and severe stunting and the potential impact of vaccination among children under five in Afghanistan using the data from the most recent Multiple Indicators Cluster Survey (MICS) in 2022-23, offering insights that are crucial for developing targeted interventions. Such localized data is vital for policymakers and health practitioners to address the unique challenges faced by Afghan children and to align strategies with the specific needs of this vulnerable population.

## Materials and methods

### Data source

This study used the data from the Afghanistan MICS 2022-23 which was conducted by UNICEF in collaboration with the National Statistics and Information Authority (NISA). The survey covers a wide range of indicators related to the situation of children and women, including child mortality, maternal and newborn health, education, water and sanitation, gender equality, and more. The survey was designed to provide estimates on a large number of indicators of the situation of children and women at the national and provincial levels [16].

### Survey sampling and participants

The MICS Afghanistan 2022-23 was conducted to provide estimates for a wide range of indicators regarding the state of women and children nationwide, in urban and rural areas, and in 34 provinces [16]. The provinces were stratified by urban and rural areas and the household sampling was carried out in two steps. In the first stage, the enumeration areas (EAs) from previous census enumeration were used as the primary sampling units (PSUs) and a specified number of EAs were selected systematically with probability proportional to size within each stratum. After a household listing was performed within the selected enumeration areas, a systematic sample of 24 households was drawn in each sample EA. The estimated sample size was 23,568 households in 982 sample enumeration areas. Questionnaires were completed for 32,989 children aged under 5, which corresponds to a response rate of 98.8% within interviewed households [16].

### Study variables

#### Dependent variable

The dependent variable was child stunting, which comprised 3 categories (no stunting, moderate stunting, and severe stunting). Child stunting refers to a child who is too short for his or her age and it was defined as the percentage of children under age 5 who fall above minus two standard deviations of the median height for age of the WHO standard for no stunting; below minus two and above minus three standard deviations of the median height for age of the WHO standard for moderate stunting; and below minus three standard deviations of the median height for age of the WHO standard for severe stunting [17].

#### Independent variables

Through a comprehensive literature review, an attempt was made to extract all the relevant and available variables for stunting from MICS 2022-23. This includes age in months (0–5, 6–11,12–23, 24–35, 36–47, 48–59); sex (male, female); area of living (urban, rural), wealth tercile; mother’s age at birth in year (≤ 18, 19–29, 30–39, 40–49); parental education level (none, primary, secondary, higher); number of children in household (1–4, 5–7, < 7).

Sanitation facility (improved, unimproved): It was considered as improved if they were designed to hygienically separate excreta from human contact and include flush/pour flush to piped sewer system, septic tanks, or pit latrines; ventilated improved pit latrines, composting toilets or pit latrines with slabs and considered unimproved if they flush to an open drain, pit latrines without a slab, hanging latrines and bucket latrines [18].

Drinking water (improved, unimproved): It was considered as improved water if collection time is not more than 30 min for a roundtrip including queuing. In addition, improved drinking water sources are those that have the potential to deliver safe water by nature of their design and construction, and include piped water, boreholes or tube wells, protected dug wells, protected springs, rainwater, and packaged or delivered water. Unimproved drinking water sources include unprotected dug wells and unprotected springs [18].

Basic vaccination: It refers to the percentage of children aged 12–35 months vaccinated against tuberculosis, polio, diphtheria, tetanus, pertussis, and measles.

Full vaccination: It refers to the percentage of children aged 24–35 months who have received all the vaccines scheduled to be given in the two first years of life, according to the national vaccination schedule.

### Statistical analysis

Descriptive statistics were employed to describe the distribution of independent variables and the prevalence of stunting across them. Chi-square analysis was used to examine the association between each independent variable with stunting. Multinomial logistic regression was used to examine the risk of stunting across different independent variables proved to be influential in bivariate analysis (*p* < 0.05). Multicollinearity was tested using the “collin” command and no VIF greater than 2 emerged. The goodness of fit for the final model was examined with the likelihood ratio test comparing the complete model with alternative nested models to choose the best model that describes the data. Separate models were constructed for basic vaccination and full vaccination as the statistical sample was restricted to children aged 12–35 and 24–35 months, respectively. Given the large sample size and high response rate, missing data were treated as missing completely at random (MCAR) and excluded from the full analysis. The results were reported as relative risk ratio (RRR) and 95% confidence interval (95%CI). The complex sampling design and sampling weights were accounted for in all analyses. The significant statistical level was set at *p* < 0.05.

## Results

### Sociodemographic characteristics

A total of 32,989 children under 5 years old were included in this study. Of those 44.7% were stunted with 21.74% being severely stunted. Approximately 20% were younger than one year and more than half (51.27%) were male. Less than a quarter (22.42%) were living in urban areas and 32.8% were of low wealth tercile households. The details of child characteristics are presented in Table 1.

### The prevalence and determinants of stunting among children under 5

The prevalence of stunting (both moderate and severe) increases with age. Children aged 24–35 months and 36–47 months showed the highest rates of severe stunting at 28.09% and 28.80% respectively. Similarly in multivariable analysis, children aged 24–35 and 36–47 months were at the highest risk of severe stunting, with RRRs of 3.93 and 3.90, respectively. Stunting was slightly less prevalent in female children (22.82% moderately stunted, 20.78% severely stunted) compared to male children (23.02% moderately stunted, 22.67% severely stunted). However, in the adjusted model, only the risk of severe stunting was lower in female children (RRR = 0.83, 95%CI: 0.77–0.89). Children living in rural areas were more prone to stunting (23.88% moderately stunted, 24.05% severely stunted) than those in urban areas (19.59% moderately stunted, 13.73% severely stunted) and were, respectively, 1.16 and 1.23 times more likely to have moderate and severe stunting. Lower wealth terciles correlated with higher stunting rates (Table 1). Children from low-income households showed the highest percentage of severe stunting (28.93%) and the risk of moderate and severe stunting was 29% and 51% lower among high-income families in the adjusted model. Mother’s younger age at birth (≤ 18 years) was associated with a higher prevalence of moderate and severe stunting (23.33% and 29.24%, respectively); however, in the adjusted model, it was only associated with severe stunting with an RRR of 0.74, 0.54, and 0.60 for age groups 19–29, 30–39, 40–49, respectively. The prevalence of stunting decreased with higher maternal education levels (*p* < 0.001). However, in adjusted models, only mothers with primary and secondary education had a lower risk of having a stunted child, particularly a severely stunted one (39% and 34% lower risk for those with primary and secondary education, respectively) as compared to no educated mothers. Similar to maternal education, higher father education was associated with a lower risk of stunting (Table 2).

**Table 1:** Prevalence of stunting and associated factors among children under 5 in Afghanistan

		No stunting	Moderately stunted	Severely stunted	
	*n* ( weighted%)	(weighted%)	( weighted%)	(weighted%)	*p*-value
		55.33	22.92	21.74	
Age (months)					
0–5	3437 (10.44)	73.89	13.96	12.15	
6–11	3167 (9.70)	75.83	14.67	9.51	
12–23	6177 (19.35)	58.64	22.68	18.69	
24–35	6342 (18.86)	45.30	26.61	28.09	
36–47	6826 (20.23)	45.79	25.41	28.80	
48–59	7040 (21.41)	52.28	25.46	22.25	< 0.001
Sex					
Male	16,841 (51.27)	54.31	23.02	22.67	
Female	16,148 (48.73)	56.41	22.82	20.78	0.004
Area of living					
Urban	4945 (22.42)	66.68	19.59	13.73	
Rural	28,044 (77.58)	52.07	23.88	24.05	< 0.001
Wealth tercile					
Low	11,797 (32.80)	46.39	24.68	28.93	
Middle	10,976 (30.05)	51.94	24.25	23.82	
High	10,216 (37.15)	65.94	20.31	13.75	< 0.001
Mother’s age at birth					
≤ 18	1658 (47.43)	47.43	23.33	29.24	
19–29	19,225 (59.64)	55.30	23.04	21.66	
30–39	9468 (29.34)	57.42	22.65	19.93	
40–49	2058 (5.52)	54.65	22.73	22.62	< 0.001
Mother’s education					
None	27,295 (78.79)	52.39	23.40	24.21	
Primary school	2490 (9.45)	62.01	24.23	13.76	
Secondary school	2441 (8.94)	68.96	18.71	12.33	
Higher	763 (2.81)	71.65	18.48	9.87	< 0.001
Father’s education					
None	17,955 (55.93)	49.20	24.14	26.65	
Primary school	3557 (13.74)	59.72	22.16	18.12	
Secondary school	6010 (20.46)	61.48	21.52	17.01	
Higher	3008 (9.88)	69.33	19.00	11.67	< 0.001
Number of Children					
1–4	11,174 (36.28)	58.65	21.90	19.45	
5–7	12,681 (37.86)	55.15	23.18	21.67	
> 7	9134 (25.86)	50.94	23.99	25.07	< 0.001
Sanitation facility					
Unimproved	17,095 (53.47)	51.65	23.92	24.44	
Improved	15,877 (46.53)	59.58	21.78	18.65	< 0.001
Drinking water					
Unimproved	13,866 (36.83)	49.96	24.53	25.50	
Improved	19,080 (63.17)	58.41	22.02	19.57	< 0.001
Basic vaccination ^a^					
No	9415 (72.11)	48.47	24.19	27.35	
Yes	3104 (27.89)	61.07	25.72	13.21	< 0.001
Full vaccination ^b^					
No	5264 (81.02)	42.90	26.14	30.96	
Yes	1078 (18.98)	55.31	28.57	16.12	< 0.001

^a^ Restricted to children aged 12–35 months (*n* = 12,519)

^b^ Restricted to children aged 24–35 months (*n* = 6342)

**Table 2:** The adjusted relative risk ratio (RRR) for stunting across potential risk factors in children under 5 in Afghanistan

	Model 1	
	Moderately stunted	Severely stunted
	RRR (95%)	RRR (95%)
Age (months)		
0–5	Reference	Reference
6–11	1.01 (0.79–1.29)	0.80 (0.62–1.04)
12–23	2.02 (1.62–2.51)*	2.05 (1.66–2.53)*
24–35	3.07 (2.52–3.75)*	3.93 (3.20–4.83)*
36–47	2.81 (2.30–3.43)*	3.90 (3.13.4.85)*
48–59	2.38 (1.94–2.93)*	2.49 (2.02–3.07)*
Sex		
Male	Reference	Reference
Female	0.93 (0.85–1.01)	0.83 (0.77–0.89)*
Area of living		
Urban	Reference	Reference
Rural	1.16 (1.00-1.34)*	1.23 (1.04–1.47)*
Wealth tercile		
Low	Reference	Reference
Middle	0.94 (0.85–1.03)	0.82 (0.73–0.92)*
High	0.71 (0.63–0.81)*	0.49 (0.42–0.57)*
Mother’s age at birth		
≤ 18	Reference	Reference
19–29	0.93 (0.77–1.14)	0.74 (0.61–0.90)*
30–39	0.83 (0.67–1.02)	0.54 (0.44–0.66)*
40–49	0.80 (0.62–1.03)	0.60 (0.46–0.77)*
Mother’s education		
None	Reference	Reference
Primary school	1.06 (0.89–1.26)	0.62 (0.50–0.78)*
Secondary school	0.79 (0.65–0.96)*	0.61 (0.48–0.77)*
Higher	0.91 (0.67–1.24)	0.66 (0.40–1.09)
Father’s education		
None	Reference	Reference
Primary school	0.85 (0.75–0.95)*	0.75 (0.64–0.87)*
Secondary school	0.85 (0.76–0.95)*	0.73 (0.64–0.83)*
Higher	0.72 (0.61–0.85)*	0.53 (0.43–0.65)*
Number of children in the household		
1–4	Reference	Reference
5–7	1.07 (0.96–1.19)	1.09 (0.98–1.22)
> 7	1.25 (1.11–1.41)*	1.44 (1.26–1.64)*
Sanitation facility		
Unimproved	Reference	Reference
Improved	0.91 (0.83-1.00)*	0.89 (0.81–0.98)*
Drinking water		
Unimproved	Reference	Reference
Improved	0.84 (0.86–1.04)	0.96 (0.86–1.06)

Base outcome: no stunting. * *p* < 0.05.

Households with more than 7 children showed the highest rates of stunting and the risk of moderate and severe stunting was, respectively, 25% and 44% higher among them compared to those with 1–4 children. Although, in bivariate analysis, improved sanitation and drinking water sources were associated with lower stunting rates (*p* < 0.001), in the adjusted model, only improved sanitation was associated with a higher risk of stunting (9% and 11% lower risk of moderate and severe stunting, respectively). Table 3 illustrates the risk of stunting among children aged 12–35 and 24–35 months for basic and full vaccination, respectively. Although in bivariate analysis (Table 1), a significant relationship was observed between stunting and both basic and full vaccination, in the adjusted model, it remained only significant among severely stunted children with 46% and 41%, a lower risk of severe stunting among children received basic and full vaccination, respectively.

**Table 3:** Vaccination status and risk of stunting among children of target age groups

	Model 2 ^a^		Model 3 ^b^	
	Moderately stunted	Severely stunted	Moderately stunted	Severely stunted
	RRR (95%)	RRR (95%)	RRR (95%)	RRR (95%)
Basic vaccination ^1^				
No	Reference	Reference	-	-
Yes	0.99 (0.87–1.13)	0.54 (0.45–0.65)*	-	-
Full vaccination ^2^				
No	-	-	Reference	Reference
Yes	-	-	1.06 (0.86–1.31)	0.59 (0.45–0.77)*

^a^ Model 1 + basic vaccination for children aged 12–35 months (*n* = 12,519). ^b^ Model 1 + full vaccination for children aged 24–35 months (*n* = 6342). Base outcome: no stunting. * *p* < 0.05

## Discussion

This study provides critical insights into the complex nature of child stunting as a critical public health challenge in Afghanistan. The prevalence of child stunting in the current study was 44.7%, which is similar to the results (46.2%) of a previous study, utilizing data from the National Nutrition Survey 2013 [6]. These figures indicate a stagnation in progress and inform the need for urgent policy efforts and interventions.

The increasing prevalence of stunting with age, particularly in children aged 24–35 and 36–47 months, aligns with previous studies indicating that the risk of stunting escalates as children grow older, possibly due to prolonged exposure to adverse factors such as inadequate nutrition and recurrent infections [19, 20]. This is compounded by the low exclusive breastfeeding rate of 58% [10], while providing some initial immunity, may not suffice for long-term nutritional needs. Additionally, the critical shortfall in neonatal and postnatal care, at a mere 19%, alongside a very low dietary diversity rate of 22% [10], suggests a nutritional deficit significant enough to perpetuate the cycle of stunting as children move past infancy.

The observed difference in stunting prevalence between genders, with a slightly lower prevalence and risk of stunting in female children, is noteworthy. This contrasts the regional trend where often female children are more likely to be stunted. For example, research in neighboring countries such as Pakistan, India, and Bangladesh consistently show a higher prevalence of stunting in female children [21, 22], while research from Sub-Saharan countries indicates a higher susceptibility of boys to stunting [23]. The disparity may be influenced by a range of factors, including household wealth, maternal literacy, food security, hygiene and sanitation conditions, and discrimination in food supply and feeding practices [21, 24]. For instance, a study in Sothern Ethiopia observed a higher prevalence of stunting among female children and suggested gender favoritism and parental partialities for male children in certain communities as potential contributing factors [25]. On the other hand, a study in Ethiopia found higher stunting in male children and attributed that to higher susceptibility to infectious diseases and higher biological fragility in the early years of life among male children [26]. A similar study in Senegal also suggested lower immune response and increased vulnerability to infections as potential causes for higher stunting rates among male children [27]. These findings suggest that both cultural and biological factors may contribute to the observed gender disparity in stunting prevalence. Culturally, the close proximity of female children to caregivers, especially during playtime and throughout the day, may result in more consistent care and oversight of feeding practices. This continuous interaction likely enhances their health outcomes due to immediate care and nutritional support when needed. Furthermore, there appears to be no significant discrepancy in the distribution of food between male and female children within Afghan households. Biologically, it has been suggested that female children may have a greater resilience to infectious diseases [26], potentially contributing to their lower rates of stunting. This inherent biological resilience, when combined with the attentive caregiving practices observed during crucial daytime hours, may be key factors in the reduced stunting rates among female children in Afghanistan. However, no previous study has described the underlying reasons among children in Afghanistan and therefore, it is essential to further investigate these factors and their impact on the gender disparity in stunting prevalence in Afghanistan. This information can help inform policies and interventions to address gender-based differences and promote gender equality in the country.

Parental education, particularly the father’s education, plays a significant role in stunting [28]. Children with less-educated parents face higher stunting rates [29–32], underscoring the importance of enhancing parental education in Afghanistan as part of stunting reduction strategies [7]. Given the restrictions on Afghan women’s education beyond the primary level, it becomes crucial to address the gap in female education, considering that mothers are often the primary caregivers of children [33, 34]. The education of mothers is essential not only for their empowerment but also for the direct impact it has on the health and nutritional status of their children. Strategies to improve child nutrition and reduce stunting should therefore include efforts to support and promote educational opportunities for both mothers and fathers, recognizing the role of maternal education in fostering early child development [35].

Another important finding in this study was a higher stunting rate in low-income households with a higher number of children (> 7). The increased risk of stunting among children under five could be due to various factors such as food insecurity, poor maternal health, insufficient feeding and care, and low household wealth [36, 37]. Research has shown that children from families with more members are at a higher risk of being stunted due to scarcity of resources for household consumption, particularly food, which could lead to stunted growth [38–40]. Additionally, household income has been highlighted as the most influential factor in predicting the food supply and influencing the incidence of stunting in children under five [41]. Thus, lower household income can also contribute to the increased risk of stunting in children from households with a higher number of children due to insufficient food supply. This scenario is further complicated by the current state of family planning and healthcare equity. With only 42% of the demand for family planning satisfied with modern methods and a 17% equity gap between the poorest and richest populations, there’s a clear indication of unequal access to essential health services [10]. This disparity is mirrored in the care-seeking behavior for symptoms of pneumonia, where a similar 17% equity gap exists between the poorest and wealthiest segments [10]. These findings underscore the interconnectedness of household income, family size, access to family planning, and equitable healthcare services in influencing child nutrition and growth outcomes. Addressing these interconnected factors through comprehensive health and nutrition programs, alongside targeted interventions to improve family planning and healthcare equity, is crucial for reducing stunting rates among children in Afghanistan.

The observed difference between rural and urban areas with respect to child stunting is consistent with previous studies, reflecting broader disparities in access to health services, nutrition, and sanitation [42, 43]. Children in rural areas are more likely to experience stunting due to factors such as limited access to healthcare, poor nutrition, and inadequate sanitation [44, 45]. Of note, we have found lower stunting among children living in households with improved sanitation. This highlights the critical role of environmental factors in children’s health. Studies have shown that children from households with unimproved WASH (water, sanitation, and hygiene) facilities, practicing open defecation, and living in households with dirt floors are more likely to be stunted [46, 47]. The difference in stunting rates between rural and urban areas in Afghanistan is a manifestation of wider gaps in healthcare [48, 49], nutrition [50], and sanitation access [51]. In rural communities, where services are often sparse and infrastructure lacking, these disparities can significantly exacerbate the incidence of child stunting. Limited healthcare access impedes early detection and treatment of conditions contributing to stunting, while poor nutrition, stemming from less diverse food availability and economic constraints, directly affects children’s growth. Additionally, inadequate sanitation facilities increase the risk of infections that can interfere with nutritional absorption, further heightening the likelihood of stunting. Such conditions in Afghanistan’s rural settings magnify the challenges of preventing and managing child stunting, underscoring the urgent need for targeted, multi-sectoral interventions to bridge these gaps and support the well-being of rural populations. However, the lack of international aid and sanctions has led to a deepening humanitarian crisis, exacerbating food insecurity and malnutrition among children [35]. It highlights the need for revising international regulations to ensure that children are not adversely affected by the broader political context, ensuring that aid reaches those most in need without hindrance.

Finally, the protective effect of basic and full vaccination against severe stunting in this study should be emphasized. This finding underscores the importance of comprehensive vaccination programs in mitigating the risk of stunting, particularly the severe type. Previous studies have shown that timely vaccinations may be an essential parameter in reducing stunting in low-income countries [52]. Malnourished children are more vulnerable to disease, which means immunization is extra important for them [53]. Childhood vaccination programs are substantially effective in reducing child mortality and protecting them from chronic cognitive and physical disabilities [54]. The COVID-19 epidemic has severely disrupted global routine child immunization programs. According to the WHO, there have been significant disruptions to regular immunization programs in numerous nations, which has a negative impact on vaccination coverage and increases the number of susceptible children, increasing the possibility of outbreaks of illnesses that can be prevented by vaccination [55]. COVID-19 had a severe impact in Afghanistan due to a lack of pandemic preparation, which included inadequate infrastructure, ongoing violence, cultural barriers, and vaccine hesitancy [56]. The increasing prevalence of waterborne and climate-sensitive diseases in Afghanistan, due to climate change, underscores the importance of (childhood) vaccination [57]. Timely vaccinations (especially among children, as most vulnerable population) become even more crucial as a protective measure against the heightened risk of diseases like diarrhea and typhoid during hotter seasons, and pneumonia during colder times of the year [57]. Moreover, there has been a decrease in international aid and funding for key healthcare initiatives, including the Sehatmandi program, which is crucial for delivering primary healthcare services across Afghanistan [58]. This reduction in support has posed significant challenges to the continuity and accessibility of essential healthcare services in the country. Additionally, recent policy changes have placed additional restrictions on women’s employment [59, 60]. These measures have affected women’s ability to attend and lead vaccination campaigns, impacting the delivery of healthcare services. Nonetheless, efforts are underway to engage with Afghanistan’s current government to facilitate the resumption of vaccination campaigns and enhance healthcare access for the Afghan population, aiming to address critical public health needs.

### Strengths and limitations

There were some strengths in our study. First, the use of data from a nationally representative sample size can increase the external validity of the study, allowing generalizability of the findings at the national level. Second, this study utilized data from MICS data set similar to those available in a number of other LMICs, and this approach has the potential that our findings would be comparable with those reported from other LMICs. Third, several correlates of child stunting do emerge, which have clear implications for policy and intervention strategies, particularly in the current sociopolitical situation of Afghanistan. However, our study has some limitations. First, some influential variables that could affect child stunting such as birth weight, birth interval, breastfeeding, and feeding practices were not available in MICS 2022-23, and thus, their effect on stunting could not be examined or accounted for in this study. Second, the data collected for the MICS were self-reported, and, therefore, are prone to information and recall bias. Finally, the cross-sectional nature of the study does not allow for any casual inferences and the observed associations should not be considered as causal, and further longitudinal studies are recommended to address them.

## Conclusion

This study underscores the necessity of a multifaceted approach in addressing stunting in Afghanistan, involving educational, nutritional, healthcare, and infrastructural improvements. It calls for concerted efforts from the international community and local governance to prioritize children’s health and well-being amidst the complex socio-political landscape of Afghanistan. The emphasis on vaccination highlights the need for continued support and adaptability in healthcare provision to navigate the challenges posed by the current sociopolitical situation. Ensuring the resilience and accessibility of healthcare services, particularly vaccination campaigns, is crucial for mitigating the impact of stunting and safeguarding the future of Afghan children.

## Data Availability

The MICS 2022-23 dataset is publicly available and is available on UNICEF’s official website through the following link: https://mics.unicef.org/surveys.
